# Prehospital Normoventilation in Critically Ill Anaesthetised Patients Cannot Be Reliably Monitored by Capnography—A Retrospective Observational Study

**DOI:** 10.1111/aas.70316

**Published:** 2026-07-29

**Authors:** Jussi Tero, Jussi Pirneskoski, Harry Ljungqvist, Juri Laamanen, Jouni Nurmi

**Affiliations:** ^1^ Emergency Medicine and Services University of Helsinki and Helsinki University Hospital Helsinki Finland; ^2^ University of Helsinki Helsinki Finland

**Keywords:** air ambulance, anaesthesia, capnography, critical care, emergency medical services, helicopter emergency medical services, intubation, prehospital, ventilation

## Abstract

**Background:**

End‐tidal carbon dioxide capnography (EtCO_2_) is widely used to guide intubated patients' ventilation. Current guidelines for prehospital advanced airway management assume a gradient of approximately 0.67 kPa between EtCO_2_ and arterial carbon dioxide partial pressure (PaCO_2_). However, concerns have been raised about the accuracy of capnography. We aimed to assess the agreement between EtCO_2_ and PaCO_2_ in different patient groups to see if EtCO_2_ could be accurate enough to guide ventilation during prehospital care.

**Methods:**

We carried out a retrospective study of all anaesthetised and intubated patients with a measured PaCO_2_ by a single helicopter emergency medical services unit from 1 January 2014 to 31 December 2021. The patients were divided into five different patient groups based on clinical diagnosis: out‐of‐hospital cardiac arrest, trauma, intoxication, neurological conditions (including stroke) and other. The agreement between EtCO_2_ and PaCO_2_ in each group was assessed with Bland–Altman analysis. In addition, the percentage of patients with a higher PaCO_2_–EtCO_2_ gradient than 0.67 kPa was calculated.

**Results:**

In Bland–Altman analysis, the overall bias was 1.7 kPa (standard deviation = 1.6) with limits of agreement of −1.4 to 4.8 kPa. Out of 670, the PaCO_2_–EtCO_2_ gradient was higher than 0.67 kPa in 530 (79.1% [75.9–82.0]) patients. 186/221 (84.2% [78.8–88.4]) in out‐of‐hospital cardiac arrest patients, 85/104 (81.7% [73.2–88.0]) in trauma patients, 146/197 (74.1% [67.6–97.7]) in neurological patients, 75/99 (75.8% [66.5–83.1]) in intoxicated patients and 38/49 (77.6% [64.1–87.0]) in other patients. The results are presented as n/nn (% [95% confidence interval]).

**Conclusions:**

Our results show that for critically ill patients intubated in the prehospital setting, capnography is not accurate in guiding ventilation.

**Editorial Comment:**

Capnography may be used to assess ventilation effect in intubated patients. In an intubated helicopter ambulance mixed‐diagnosis cohort end‐tidal carbon dioxide levers were assessed, compared to pCO_2_ results from a point of care blood gas analysis device. The proportion of larger gaps between these two values in this mixed cohort is presented.

## 1 Background

In patients undergoing prehospital emergency anaesthesia (PHEA), normoventilation is crucial in many patient groups. Especially for patients suffering from traumatic brain injury (TBI), as hypo‐ or hypercapnia may alter intracranial perfusion, causing possibly preventable secondary damage to the brain. Both hypocapnia and hypercapnia have been shown to be associated with increased mortality and worse neurological outcome in TBI [1, 2, 3, 4]. End‐tidal carbon dioxide capnography (EtCO_2_), which measures carbon dioxide from the exhaled gas at the end of expirium and displays a continuous waveform and a numerical value, is used to guide ventilation in the prehospital setting since it has been thought to underestimate arterial CO_2_ only slightly.

With its easy non‐invasive modality, EtCO_2_ proposes a tempting surrogate for arterial carbon dioxide partial pressure (PaCO_2_), and based on earlier studies, the PaCO_2_–EtCO_2_ gradient has been estimated to be approximately 0.5 kPa. However, this approximation is mainly based on healthy anaesthetised in‐hospital patients, and recent literature indicates that severely ill patients anaesthetised in a prehospital setting may have a greater and more unpredictable PaCO_2_–EtCO_2_ gradient than suggested [1, 5, 6, 7, 8, 9, 10]. Current guidelines for prehospital advanced airway management suggest that EtCO_2_ and PaCO_2_ should be between 4 and 6 kPa and 4.67 and 6.67 kPa, respectively, when managing critically ill intubated patients in the prehospital setting. However, tighter ventilation control, with EtCO_2_ between 4 and 4.67 kPa and PaCO_2_ between 4.67 and 5.33 kPa, is recommended for patients suffering from TBI. These recommendations assume a PaCO_2_–EtCO_2_ gradient of 0.67 kPa [11].

We have recently described a prevalence of a high PaCO_2_–EtCO_2_ gradient in PHEA patients [7]. However, it is unknown what proportion of the patients falls within the PaCO_2_–EtCO_2_ gradient of 0.67 kPa, suggested by the recent quality indicators, or if the gradient differs between patient groups. Consequently, in the current study with an extended patient cohort in addition to the previous paper, we aimed to determine the bias and limits of agreement between PaCO_2_ and EtCO_2_ in five different patient groups based on the clinical diagnosis by a helicopter emergency medical services (HEMS) physician. We also aimed to determine the proportion of patients with PaCO_2_–EtCO_2_ gradients exceeding 0.67 kPa.

## 2 Methods

### 2.1 Study Design

We carried out a retrospective observational registry study of all patients anaesthetised and intubated by a single HEMS unit from 1 January 2014 to 31 December 2021.

Since this was a retrospective registry‐based study, ethical approval was not required according to Finnish legislation. Access to patient records was granted by the Helsinki University Hospital (HUS/305/2022). This study was reported according to Strengthening the Reporting of Observational studies in Epidemiology (STROBE) guidelines [12].

### 2.2 Setting

The HEMS unit, FinnHEMS 10, operates in the Southern Finland area and serves a population of approximately 1.3 million. It is one of Finland's seven nationally operated HEMS. In the operating area, it provides the majority of PHEAs, while a small minority of PHEAs are provided by locally operating ground‐based physician‐staffed units. In the HEMS unit, PHEA is highly standardised and regularly trained. Clinical governance procedures cover all PHEA cases. The protocol and its implementation have been recently described in detail [13, 14]. The unit is staffed by an on‐call physician specialised in anaesthesiology, with a crew consisting of a HEMS paramedic and a pilot. The unit responds to missions with critically ill or seriously traumatised patients with a ground ambulance team to provide prehospital critical care and, if appropriate, to transport the patient by helicopter.

Arterial blood gas (ABG) analyses are conducted at the on‐call physician's discretion using a point‐of‐care (POC) device, which provides the results on scene. POC devices used during the study period were either iSTAT 1 (Abbott, IL, USA) until January 2018 or iSTAT Alinity (Abbott) from January 2018 onwards. After analysis, the data is transferred to the electronic patient record system (Merlot Medi, CGI Suomi, Finland) manually.

Monitoring of vital signs and continuous EtCO_2_ is carried out with a monitor–defibrillator. Either a device of the HEMS unit or a ground ambulance is used. The devices used were either Lifepak 15 (Physio‐Control, WA, USA), CorPuls3 (Corpuls, Germany) or Zoll X series (Zoll Medical Corporation, MA, USA). The monitor–defibrillator data is automatically transferred to the electronic patient record via WiFi or Bluetooth. An Oxylog 3000 plus ventilator (Dräger, Germany) was used to ventilate intubated patients until December 2016, and a Hamilton‐T1 ventilator (Hamilton Medical, Switzerland) afterwards.

### 2.3 Participants

All patients who were anaesthetised and intubated by the HEMS unit during the study period were eligible for this study and, therefore, included in the dataset. We excluded patients without blood gas analysis results after the intubation and patients intubated during out‐of‐hospital cardiac arrest (OHCA). Patients intubated after return of spontaneous circulation (ROSC) were included in the study. In theory, a single patient may have been encountered and treated with PHEA by the HEMS unit during the study period more than once. These cases were not excluded, as the acute condition likely affects the PaCO_2_–EtCO_2_ gradient more than the underlying conditions. In cases of multiple arterial samples analysed during prehospital care, only the first one was included in the study. Power analysis or sample size calculations were not performed prior to data collection.

We divided the patient population into five subgroups based on the primary clinical diagnosis made by the on‐call physician at the scene. These included ‘OHCA’, ‘Trauma’, ‘Intoxication’, ‘Neurological conditions’ and ‘Other’. ‘OHCA’ included patients intubated after ROSC and ‘Trauma’ included all traumatised patients, as well as TBI patients. ‘Neurological conditions’ included all patients with acute neurological pathology such as stroke, status epilepticus and non‐traumatic intracranial haemorrhage. The classification was based on the international consensus‐based guidelines on data collection [15]. However, the smallest patient groups were combined as ‘Other’ for purposeful analysis and can include groups such as ‘breathing difficulties’, ‘myocardial infarction’ or ‘chest pain’, ‘obstetric’, ‘infection’, ‘asphyxiation’ or ‘anaphylaxis’.

### 2.4 Variables

The primary endpoint variable was bias and limits of agreement between PaCO_2_ and EtCO_2_ in every patient group. The secondary endpoint was the proportion of patients with PaCO_2_–EtCO_2_ gradients exceeding 0.67 kPa.

All the ABG analyses were obtained after intubation and before hospital arrival. The analyses were manually paired with the closest EtCO_2_ value in the prehospital patient record system. A maximum of a 5‐min difference between the time of ABG and EtCO_2_ was accepted to include the patient in the study.

### 2.5 Data Sources

We extracted data from the FinnHEMS database (FHDB), a national electronic database including detailed structured data from all Finnish HEMS missions. Most of the details in the FHDB are entered manually by the physician on‐call promptly after a HEMS mission. The data follows an international standardised template [16]. The blood gas results and concurrent capnography values were collected retrospectively through manual chart review of electronic prehospital records.

### 2.6 Statistical Methods

Continuous variables are presented as mean and standard deviation (SD) or median and first quartile and third quartile [IQR] based on the data distribution, assessed visually using histograms. Categorical data are presented as number and percentage (%). We calculated 95% confidence intervals (95% CIs) for proportions using the modified Wald method. To assess the agreement between PaCO_2_ and EtCO_2_, we constructed Bland–Altman plots of the variables. The results are presented as bias and limits of agreement. Bland–Altman analysis was chosen for its suitability for method comparison, providing an assessment of the agreement, systematic bias, and consistency between the two measurement methods. Analyses were performed using SPSS Statistics for Mac, version 28 (IBM Corp., Armonk, NY, USA) and GraphPad Prism for Mac, version 9 (Dotmatics, Boston, MA, USA).

## 3 Results

**FIGURE 1:**
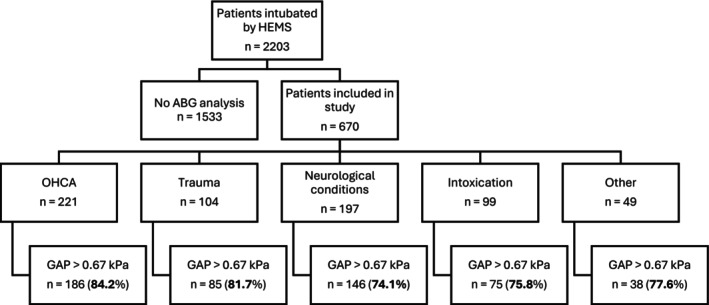
Patient selection flowchart. ABG, arterial blood gas analysis; GAP, PaCO_2_–EtCO_2_ gradient; HEMS, helicopter emergency medicine services; OHCA, out‐of‐hospital cardiac arrest.

Out of 2203 patients who were anaesthetised and intubated by the HEMS unit during the study period, 670 (30.4%) had an ABG analysis performed and were included in the analysis (Figure 1). No exclusions were made based on patient characteristics or demographics. Patient characteristics are presented in Table 1.

**TABLE 1:** Patient characteristics.

	Missing data, *n*	All patients (*n* = 670)	OHCA (*n* = 221)	Trauma (*n* = 104)	Neuro (*n* = 197)	Intoxication (*n* = 99)	Other (*n* = 49)
Sex, male	3	435 (65.2)	166 (75.5)	80 (76.9)	112 (57.1)	50 (51.0)	27 (55.1)
Age, years	0	58 (42–69)	63 (52–71)	52 (33–64)	63 (50–71)	39 (28–50)	58 (35–70)
Vitals at HEMS arrival							
Heart rate, bpm	79	95 (76–118)	104 (84–125)	88 (68–110)	93 (74–114)	93 (69–113)	110 (95–129)
Systolic blood pressure, mmHg	96	134 (115–162)	129 (108–150)	131 (118–153)	152 (129–190)	120.5 (102–138)	130 (100–162)
Oxygen saturation, %	105	97 (93–99)	97 (89–99)	97 (93–98)	97 (94–99)	98 (96–100)	93 (81–97)
Glasgow Coma Scale score	9	3 (3–6)	3 (3–3)	5 (3–8)	4 (3–6)	3 (3–6)	4 (3–8)

*Note:* Continuous variables are presented as median and interquartile range (25th–75th percentile); categorical variables are presented as number and percentage (%).

Abbreviations: HEMS, helicopter emergency medical services; Neuro, neurological conditions; OHCA, out‐of‐hospital cardiac arrest.

**FIGURE 2:**
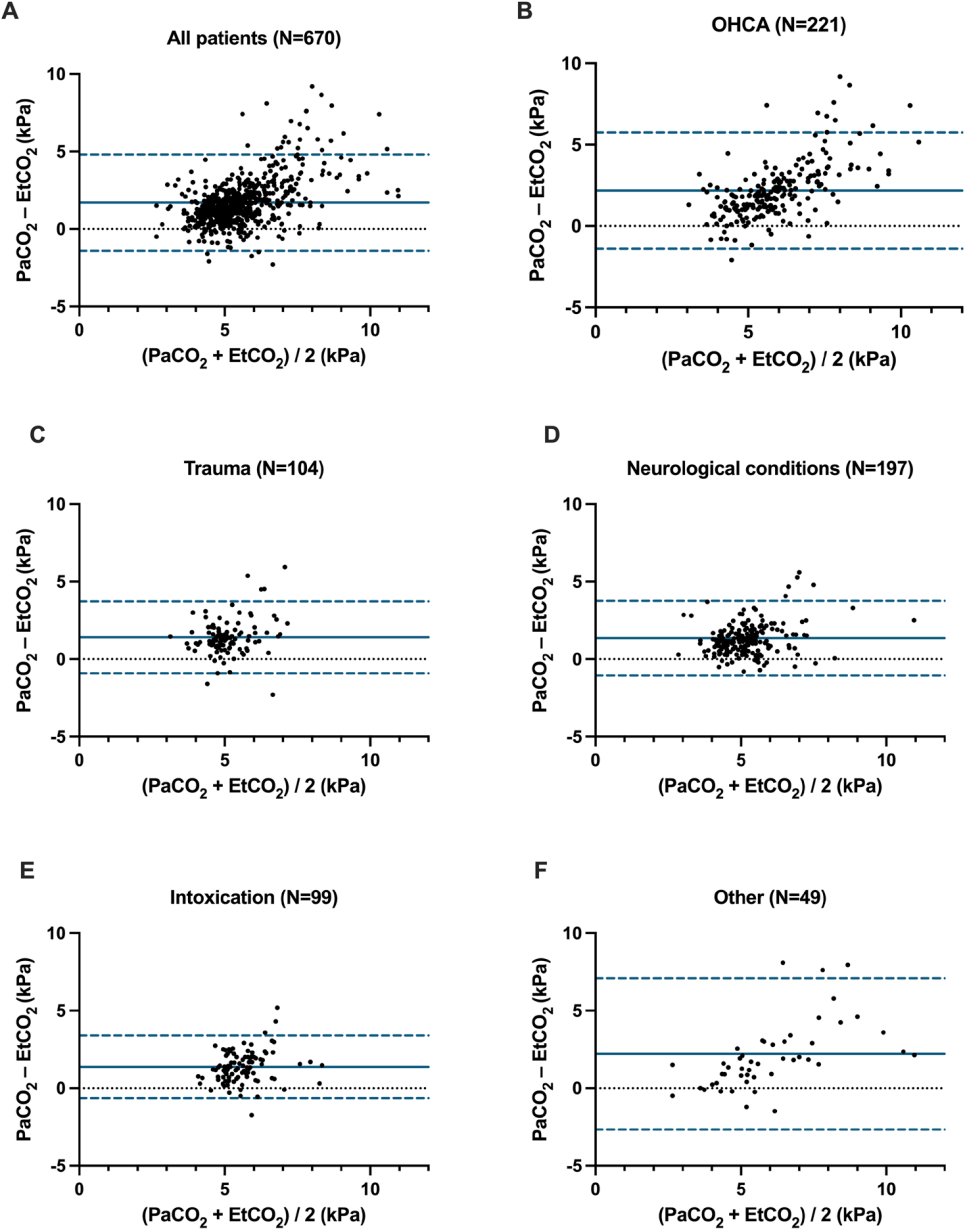
Bland–Altman analyses. Patient groups are presented as separate panels. (A) Whole patient population; (B) OHCA (out‐of‐hospital cardiac arrest); (C) Trauma; (D) neurological conditions (including stroke); (E) intoxication; (F) other. Dotted lines present 95% limits of agreement. Continuous line presents bias. PaCO_2_ and EtCO_2_ in kPa.

In every patient group, the observed PaCO_2_–EtCO_2_ gradient exceeded 0.67 kPa in the majority of patients, with the bias ranging from 1.3 (1.2) to 2.2 (2.5) kPa. Bias was higher in groups ‘OHCA’ and ‘Other’, compared to other groups, and seemed to be higher along with increasing overall CO_2_. Limits of agreement were large across the board as well. The results of the Bland–Altman analyses are shown in Figure 2.

In addition, the PaCO_2_–EtCO_2_ gradient was larger than 0.67 kPa in 530 (79.1%) patients. Table 2 presents the PaCO_2_ and EtCO_2_ values, along with limits of agreement. Overall, the bias was 1.7 (1.6) kPa and the limits of agreement between PaCO_2_ and EtCO_2_ ranged from −1.4 to 4.9 kPa.

**TABLE 2:** End‐tidal (EtCO_2_) and arterial (PaCO_2_) CO_2_ partial pressures, and bias and 95% agreement between the measurement methods, during prehospital anaesthesia.

	All patients (*n* = 670)	OHCA (*n* = 221)	Trauma (*n* = 104)	Neuro (*n* = 197)	Intoxication (*n* = 99)	Other (*n* = 49)
EtCO_2_, kPa	4.72 (1.04)	4.83 (1.06)	4.41 (0.84)	4.57 (1.00)	4.95 (0.84)	5.01 (1.52)
PaCO_2_, kPa	6.42 (1.83)	7.01 (2.11)	5.82 (1.09)	5.92 (1.48)	6.32 (1.03)	7.22 (2.95)
> 0.67 GAP, *n* (%)	530 (79.1 [82.0–75.9])	186 (84.2 [78.8–88.4])	85 (81.7 [73.2–88.0])	146 (74.1 [67.6–79.7])	75 (75.8 [66.5–83.1])	38 (77.6 [64.1–87.0])
Bias, kPa	1.7 (1.6)	2.2 (1.8)	1.4 (1.2)	1.3 (1.2)	1.4 (1.0)	2.2 (2.5)
95% Limits of agreement, kPa	−1.4 to 4.8	−1.4 to 5.8	−0.9 to 3.7	−1.1 to 3.8	−0.7 to 3.4	−2.7 to 7.1

*Note:* The data are presented as mean (SD) and *n* (% [95% confidence interval]).

Abbreviations: Neuro, neurological conditions; OHCA, out‐of‐hospital cardiac arrest.

## 4 Discussion

### 4.1 Main Findings

In this retrospective study of prehospital anaesthetised patients, we found that the bias between PaCO_2_ and EtCO_2_ exceeded 1.2 kPa and limits of agreement were substantially wide in all patient groups, with the vast majority of patients having a higher PaCO_2_–EtCO_2_ gradient than proposed by the prehospital anaesthesia quality indicators [11]. Bias increasing with overall CO_2_ shows that the PaCO_2_–EtCO_2_ gradient is not constant across different EtCO_2_ values. The findings strongly suggest that normoventilation is not achievable using solely EtCO_2_ to guide ventilation in any patient group undergoing PHEA.

### 4.2 Clinical Relevance

Our results show that clinical diagnosis has limited predictive value when assessing the need for ABG analyses in adjusting ventilation, and end‐tidal capnography alone may expose patients to hypo‐ or hypercapnia. Hypocapnia reduces cerebral blood flow (CBF) and thus might cause ischemia, resulting in secondary brain injury in neurocritical patients. Hypercapnia, on the other hand, increases CBF, leading to higher ICP, possibly worsening existing haemorrhage or causing herniation [17].

This phenomenon is especially important in critically ill patients undergoing PHEA, since they might suffer from primary brain injury and brain oedema from inadequate oxygenation or trauma [18]. Even relatively small deviations from normocapnia might have major consequences when intracranial pressure is already elevated. In patients with TBI, worse outcomes have been demonstrated in both hypoventilated and hyperventilated patients compared to normoventilated patients. This association is observed only in intubated patients but not in patients breathing spontaneously [2]. The association between prehospital normoventilation and better outcomes of patients intubated in a prehospital setting has been confirmed in multiple studies [1, 2, 3, 9], which, along with the CO_2_ reactivity of CBF, provide pathophysiologic support for maintaining normoventilation to limit secondary brain injury. The current evidence relies mainly on EtCO_2_ levels, and accurate blood CO_2_ levels by ABG analyses during the prehospital phase have not been studied. As EtCO_2_ is quite unreliable during prehospital care, more evidence of ventilation guided by PaCO_2_ is needed.

The literature is mainly focused on TBI patients, but other critically ill patients might suffer from inadequate ventilation as well. Since most of the patients prior to intubation in the prehospital setting might have insufficient perfusion to the brain, inadequate physiologic regulation of CBF or elevated ICP, they are susceptible to secondary brain injury regardless of the clinical diagnosis. In addition, hypercapnia might worsen acidosis in critically ill patients. Thus, even without a TBI, the deviation from normocapnia should be avoided. Similarly to our results, other studies have also reported high PaCO_2_–EtCO_2_ gradients with moderate correlation at best, which makes ventilation based on capnography questionable [1, 7]. Studies have found a large PaCO_2_–EtCO_2_ gradient at hospital arrival when treating patients with TBI [8, 9]. In OHCA patients achieving ROSC, similar results have been reported [19, 20].

Several factors may contribute to the difference between PaCO_2_ and EtCO_2_. This difference is primarily caused by ventilation‐perfusion mismatch, where circulating blood does not reach ventilated alveoli for gas exchange, or reaches alveoli that are not ventilated. In healthy individuals, this mismatch, and therefore the PaCO_2_–EtCO_2_ gradient, should be small. However, it can be increased by hypotension, as blood pressure may not be sufficient to maintain adequate pulmonary circulation, resulting in reduced gas exchange [21]. Another cause might be inadequate ventilation and atelectasis, as blood circulation perfuses closed, unventilated alveoli. Higher tidal volumes and lower ventilatory frequency might lower mismatch between pulmonary ventilation and circulation, thereby lowering the PaCO_2_–EtCO_2_ gradient [6].

The reasons behind the higher bias in the ‘OHCA’ and ‘Other’ groups in our Bland–Altman analyses are not certain. One can speculate that patients suffering OHCA may have been hypoventilated during chest compressions, and aspiration is likely more common in this group. The ‘Other’ group includes patients with respiratory failure as the main problem among many other heterogeneous preliminary diagnoses, which do not allow for a more precise explanation based on the current data.

The PaCO_2_–EtCO_2_ gradient is, however, impossible to estimate accurately without an ABG analysis, and PaCO_2_ could be adjusted with ventilation optimisation if an ABG analysis was available to guide treatment. The fact that arterial cannulation doesn't seem to delay definitive treatment further encourages obtaining an ABG during prehospital treatment [22].

### 4.3 Limitations

Our study has several limitations that need consideration. First, it was retrospective and was limited to the extent of data already gathered. The PaCO_2_ from the ABG analysis was not compared with the capnography at the exact same time, thus possibly resulting in measurement error in the PaCO_2_–EtCO_2_ gradient. But as we limited the maximum time difference to 5 min and the vital signs are automatically recorded, we estimate this error to be minimal.

Second, the time between intubation and the ABG analysis is not reliably available from the database. This could lead to a falsely high PaCO_2_–EtCO_2_ gradient since it might be higher immediately after intubation compared to a period of mechanical ventilation and haemodynamic optimisation. However, the extent of this is unclear, since our results are in line with other studies that have measured ABGs at hospital arrival [8, 9, 19, 20].

Third, since ABG analysis is not performed on every patient, there is a risk of selection bias towards a more morbid population of patients with possibly higher PaCO_2_–EtCO_2_ gradients. However, 30% of all anaesthetised patients during the study period were included in the study, and almost 80% of these had a higher PaCO_2_–EtCO_2_ gradient than 0.67 kPa. This indicates that despite the possible selection bias, the results represent a significant proportion of all anaesthetised patients.

Finally, the patients included in the study represent a highly heterogeneous group. The classification of the patients into groups by the prehospital physician is not fully standardised and may lead to some patients being placed in the wrong category based on the initial evaluation, which could cause bias. However, we estimate this to be of no major relevance, as a previous study on the reliability of the database showed that the inter‐rater reliability of the classification appears to be rather high [23]. The data collection period was several years, during which different monitor–defibrillators were used. The influence of this on measured EtCO_2_ values cannot be ruled out.

## 5 Conclusions

The gradient between EtCO_2_ and PaCO_2_ is large and unpredictable during anaesthesia of critically ill prehospital patients. Consequently, relying exclusively on EtCO_2_ to achieve normoventilation is likely to be unreliable.

## Author Contributions

J.T. and J.P. prepared the first version of the manuscript. J.N., J.L. and H.L. gathered, and all authors interpreted the data. H.L. and J.N. performed the statistical analyses. J.T. and H.L. prepared Tables 1 and 2. J.N. prepared Figure 2. J.P. and J.N. supervised the study process. All authors made significant contributions to the concept and design of the study as well as to the revision and editing of the manuscript. All authors have read and approved the final manuscript.

## Funding

This work was supported by Helsingin ja Uudenmaan Sairaanhoitopiiri (VTR TYH2022320).

## Ethics Statement

The authors have nothing to report.

## Consent

The authors have nothing to report.

## Conflicts of Interest

The authors declare no conflicts of interest.

## Data Availability

An anonymised dataset can be made available upon a reasonable request from the corresponding author.
